# Draft Genome Sequence of an Aspergillus Strain Isolated from a Honey Bee Pupa

**DOI:** 10.1128/mra.00798-22

**Published:** 2022-10-04

**Authors:** E. Anne Hatmaker, Delaney L. Miller, Irene Newton, Antonis Rokas

**Affiliations:** a Department of Biological Sciences, Vanderbilt University, Nashville, Tennessee, USA; b Vanderbilt Evolutionary Studies Initiative, Vanderbilt University, Nashville, Tennessee, USA; c Department of Biology, Indiana University, Bloomington, Indiana, USA; University of California, Riverside

## Abstract

Insect-associated fungi play an important role in wild and agricultural communities. We present a draft genome sequence of an entomopathogenic strain from the fungal genus Aspergillus, isolated from a honey bee pupa.

## ANNOUNCEMENT

Fungi are a leading cause of insect disease (1) and negatively impact the productivity of an economically and ecologically important pollinator, the honey bee (2–4). The risk of fungal disease is greatest for honey bee brood (larvae and pupae). Several species of Aspergillus are opportunistic brood pathogens (5). Aspergillus species also infect plants (6) and humans (7).

We isolated a fungal strain (DMIN) from an infected pupa in Bloomington, IN, in June 2018. Inoculation of *in vitro*-reared bee brood with 10^3^ fungal spores confirmed the pathogenic potential of the strain (8). Spores were collected with a sterile swab and struck to isolation on potato dextrose agar (PDA) at 34°C, ambient humidity, in incubation chambers under conditions favorable for microaerophile growth. Total DNA was extracted with the DNeasy blood and tissue kit (Qiagen, Hilden, Germany). The isolate ITS region was amplified using ITS4/ITS5 (9) and compared to the NCBI nonredundant/nucleotide (nr/nt) database using BLASTN (10); the region was most similar (>90%) to multiple Aspergillus species, with 100% query coverage.

Libraries were prepared using the TruSeq DNA PCR-free kit (Illumina, Inc.), and genomic DNA (gDNA) was sequenced at the Vanderbilt Technologies for Advanced Genomics facility on the Illumina NovaSeq 6000 platform, resulting in 150-bp paired-end reads. The reads were filtered for quality and adaptors removed using Trimmomatic v0.39 (11). The trimmed reads were assembled using SPAdes v3.15.3 (12). The mitochondrial genome was assembled using GetOrganelle v1.6.4 (13) and annotated using GeSeq v2.03 (14, 15). The nuclear genome was annotated using Liftoff v1.2.0 (16) with Aspergillus flavus NRRL 3357 as the reference genome (GenBank accession number GCA_000006275.3) (17). We evaluated the genome completeness using the BUSCO v4.0.4 Eurotiales database (18). The reads were mapped to assemblies using Bowtie2 v2.3.4.1 (19). The average nucleotide identity (ANI) was calculated using FastANI v0.1.3 (20). ANI comparisons were made between DMIN and four *Flavi* species: Aspergillus arachidicola CBS 117612 (GCA_009193545.1), Aspergillus flavus NRRL 3357 (CP044616 to CP044623), Aspergillus parasiticus SU-1 (GCA_000956085.1), and Aspergillus minisclerotigenes CBS 117635 (GCA_009176455.1). For all analyses, default parameters were used except where otherwise noted.

Sequencing resulted in 53,363,706 paired reads (42,289,484 after trimming). The assembly revealed a coculture, with both bacterial and fungal scaffolds. The fungal scaffolds were longer, had higher coverage (over 200×), and had a lower GC content (48%) than the bacterial scaffolds (coverage, 40×; GC content, 60%). Scaffolds under 500 bp were removed from the assembly, and scaffolds 500 bp and longer were compared to the NCBI BLASTN nonredundant/nucleotide (nr/nt) database. Scaffolds with high similarity (≥90%) to Aspergillus species and with ≥80% query coverage were retained to ensure that the assembly represented only fungal scaffolds. Of the 6,156 scaffolds removed, 5,281 were under 1,000 bp; the mean length of the removed scaffolds was 1,111.78 bp, and the median length was 641 bp.

ANI comparisons with Aspergillus section *Flavi* species showed that DMIN was most closely related to Aspergillus parasiticus (98% similarity). DMIN shared 96.5%, 94.2%, and 94% identity with *A. arachidicola*, *A. minisclerotigenes*, and A. flavus, respectively. The Aspergillus sp. strain DMIN nuclear genome contained 244 scaffolds; the mitochondrial genome was a single, circular chromosome (Table 1). Predicted proteins encompassed 93% of the BUSCO single-copy orthologs (3,911), with 5% (223) missing.

**TABLE 1:** Genome sequencing and assembly statistics for the bacterial and fungal genomes from isolate DMIN

Species	Genome	No. of scaffolds	*N*_50_ (bp)	Coverage (×)	Size (bp)	No. of mapped reads (%)	No. of predicted proteins	GenBank accession no.
Aspergillus sp. DMIN	Nuclear	244	14	290	39,974,781	96.22	13,169	JALMFT000000000
	Mitochondrial	1	1	3,100	29,141	1.52	15	CM042804
Bombella sp. DMIN-2	Bacterial	21	4	45	2,057,252	0.77	1,883	JAMWFD000000000

Reads that did not map to the fungal assembly were assembled independently to capture the genome of the bacterial contaminant and annotated using PGAP v6.0 (21). BLASTN comparisons of the bacterial scaffolds indicated high similarity (99%) to *Bombella* sp. strain ESL0368, which is related to bacteria that can provide protection against fungal infection in honey bee brood (8). The *Bombella* DMIN-2 assembly contained 21 scaffolds (Table 1).

Understanding Aspergillus pathogenicity is crucial to protecting insect health. The Aspergillus sp. DMIN genome provides valuable information about the genetic content of an entomopathogenic strain belonging to a widespread fungus genus.

### Data availability.

The metadata and complete assemblies are available at GenBank under BioProject accession number PRJNA815363. The raw sequence reads have been deposited in the Sequence Read Archive (SRA) under accession number SRR18306612. The Aspergillus sp. DMIN whole-genome shotgun sequencing project has been deposited at DDBJ/ENA/GenBank under the accession number JALMFT000000000. The version described in this paper is version JALMFT010000000. The Bombella sp. DMIN-2 whole-genome shotgun sequencing project has been deposited at DDBJ/ENA/GenBank under the accession number JAMWFD000000000. The version described in this paper is version JAMWFD000000000.
